# Clinical Ophthalmic Outcomes and Impact of Single Large-Scale Mitochondrial DNA Deletions

**DOI:** 10.3390/jcm14082537

**Published:** 2025-04-08

**Authors:** Michael Otakhor Erhunmwunse, Pushpa Raj Joshi

**Affiliations:** 1 Department of Biotechnology, Brandenburg University of Technology, 03046 Cottbus-Senftenberg, Germany; erhunmic@gmail.com; 2 Institute of General and Family Medicine, Martin-Luther University Halle-Saale, 06112 Halle (Saale), Germany

**Keywords:** mitochondrial DNA, CPEO, ptosis, common deletion (CD)

## Abstract

**Introduction/Objectives**: Chronic progressive external ophthalmoplegia (CPEO) is commonly associated with mtDNA deletions. Multiple deletions result mostly due to nuclear DNA defects that lead to an autosomal mode of inheritance, whereas single mtDNA deletions are mostly sporadic events with low inheritance risk. The study focused on assessing the clinical ophthalmic outcomes and their effects on patients with mitochondrial DNA disorders. **Methods**: A retrospective analysis of clinical characteristics in a cohort of CPEO patients (n = 36; 11 males, 25 females; mean age of onset: 41.2 years (±SD)) was performed. The underlying genetic defects, as well as histological features and their correlation with the clinical features, were evaluated. **Results**: Ptosis (56% of patients) was a frequently identified clinical symptom. Single mtDNA deletions were reported in all patients, and the ‘common’ 4977 bp deletion (CD) was detected in 11 patients (30.6%). The incidence of the common deletion was higher (36.36%) in older patients (≥51 years) as compared to younger patients (18.18%). The mean age of onset in patients harboring CD was 27 years (±11.9). Furthermore, a tendency to increase the frequency of COX-deficient fibers with increasing age was observed in patients harboring the CD. **Conclusions**: The present study shows that CD is typically associated with elderly patients with CPEO. Moreover, ptosis and the presence of a single deletion in patients with mitochondrialopathy seem to be preliminary diagnostic criteria.

## 1. Introduction

Many efforts have been made toward identifying mtDNA disease treatment, but these have been only partially successful, and the possible therapies are not completely effective for patients with mitochondrial disorders [1].

An alteration in the nucleotide sequence of the human mitochondrial DNA (mtDNA) stands as an important cause of genetic diseases [2], and these alterations have long been implicated in the physiological changes within aging skeletal muscle [3] and human diseases. Mitochondrial diseases constitute a group of metabolic disorders with heterogeneous symptoms. Furthermore, the mitochondrial genome is said to be dysfunctional when the copy number of the mutant mtDNA has exceeded a certain threshold level of threshold within an individual cell, which is known as the threshold effect [4]. It is also called the minimum critical number required to cause mitochondrial disorders [5]. However, this threshold level has been proposed to be in varying dimension rates as regards individual cells and tissue types. A percentage concentration of normal mtDNA between 10 and 30% has been proposed to be sufficient to compensate for the presence of any mutant mtDNA, while a threshold level between 70% and 90% of mutant mtDNA has been investigated to be associated with clinicopathological characteristics [6]. However, a research study carried out by Procaccio et al. (2006) on a few type II diabetic patients harboring mitochondrial disorders revealed a low percentage level of mtDNA point mutations [7].

The classical trait of mitochondrial disorders is the presence of heterogeneous clinical symptoms associated with a unique genetic variant [8]. Patients with mtDNA disorders are usually presented with a few major characteristics and phenotypes that include CPEO, Kearns–Sayre Syndrome (KSS), Pearson Syndrome, and bilateral ptosis [9,10]. Moraes et al. (1989) [11] reported that CPEO and bilateral ptosis, which could be associated with a high concentration of protein level in the CSF in younger patients with mtDNA deletion, can cause a clinical term called KSS [12]. However, groups of families having the same genetic defects can surprisingly manifest different clinical characteristics, a phenomenon that is yet to be understood. The deletion of the mtDNA at a base pair (bp) from 2 kb to 10 kb presents a few mitochondrial disorders, collectively called mtDNA deletion syndrome, which are clinically interrelated [13].

A common mitochondrial clinical feature, **CPEO**, is characterized by gradually worsening bilateral ptosis and ophthalmoplegia. It may present as the sole clinical feature in patients with isolated ocular myopathy or in combination with neuromuscular disease manifestations (CPEO plus) [14]. CPEO is frequently associated with the loss of a significant portion of the mitochondrial genome, which can result from single large-scale mtDNA deletions or nuclear gene defects that disrupt mtDNA maintenance, leading to multiple mtDNA deletions [15]. Moreover, CPEO is clinically defined by the presence of retinitis pigmentosa associated with onset generally less than 20 years of age, with a significant relationship between deletion size and transfer RNA (tRNA) [16,17]. Apart from CPEO, patients with mtDNA disorders also present with a spectrum of clinical phenotypes that include Kearns–Sayre Syndrome (KSS), Pearson Syndrome, and bilateral ptosis [18,19].

Histological diagnostics that are commonly associated with mtDNA disease using muscle biopsies are the detection of ragged-red fibers (RRF) by Gomori trichrome that stains positive succinate dehydrogenase (SDH)m and negative to cytochrome-c-oxidase (COX) [20]. On the skeletal muscles of patients with mitochondrial myopathy, these stains could present a low level or absence of COX activity [21]. Zeviani and other researchers, in 1988, did a study on patients with KSS and observed the accumulation of abnormal mitochondria in the subsarcolemmal membrane of the mitochondria, which resulted in scattered fibers called RRFs [22]. The abnormal accumulation of mitochondria around the edge of muscle fibers (RRFs) is proposed to be an important histological biomarker of mtDNA disease, and a large number of blue (COX-negative, SDH-positive) muscle fibers is a diagnostic feature of mitochondrial myopathy caused by mtDNA mutations.

The present study investigates the clinical ophthalmic outcomes and impact of mtDNA single deletions in patients and their association with clinical features. This is to enable researchers to understand the clinical, histological, and molecular genetics aspects in skeletal muscle tissues of patients suspected to harbor mtDNA single large-scale deletions. Two or more of these findings widen the scope of adequate investigations of mtDNA diseases in patients.

### Patients

Clinical characteristics and biochemical features in a cohort of 36 patients (11 males, 25 females; mean age of onset: 41.2 years (±SD)) with molecular genetically confirmed single mtDNA deletion were analyzed. Written consent was received from all patients and the legal guardians of minors. Clinical data of the patients were extracted retrospectively from their respective records from the archive of the Department of Neurology, Martin-Luther University Halle-Wittenberg. Detailed epidemiological and clinical data of the patients are listed in Table 1. The mean age of disease onset was 27 years (range: 10–53 years). The thirty-six index patients in this study were grouped into two age groups: ≤18 years and >18 years (Table 2). Moreover, based on clinical characteristics, the patients were further divided into two sub-groups: patients with pure CPEO (n = 9) and patients harboring CPEO along with other symptoms (CPEO plus) (n = 27) (Table 2).

## 2. Materials and Methods

### 2.1. Histological Analysis

Skeletal muscle tissues were obtained in open biopsy and stored in liquid nitrogen for further analysis (n = 17). Transverse cryostat sections were cut at 5 or 10 μm thickness and stained according to modified standard protocols. Muscle biopsies of 19 patients were not available for histological studies, and the analysis was based on clinical and molecular genetic analysis. A major number of patients had CPEO and Ptosis as clinical manifestations (Table 1). Muscle biopsies were frozen with immediate immersion in methyl-butane until the bubbling stopped, and transverse cryostat sections were cut at 5 or 10 μm thickness. The Gomori trichrome staining (GTS) technique was performed with sequential cytochrome-c-oxidase and succinate dehydrogenase (COX/SDH) enzyme histochemistry according to modified standard protocols.

#### COX/SDH Stain

Air-dried cryostat sections (10 μm) were incubated in COX medium (20 mg cytochrome c (Sigma-Aldrich, St. Louis, MO, USA), 10 mg diaminobenzidine (DAB) tetrahydrochloride (Sigma-Aldrich) and 2 mL 0.001% catalase (Serva Electrophoresis, Heidelberg, Germany) in 9 mL 0.05 mol/L phosphate buffer, pH 7.4 (Sigma-Aldrich)) at 37 °C for 1 h. The excess medium was washed off with 0.05 mol/L phosphate buffer (pH 7.4). Subsequently, the sections were incubated in SDH medium ((800 μL 1.875 mmol/L nitroblue tetrazolium (Sigma-Aldrich), 100 μL 130 mmol/L sodium succinate (Sigma-Aldrich), 100 μL 2 mmol/L phenazine methosulphate (Serva Electrophoresis) and 10 μL 100 mmol/L sodium hydroxide (Sigma-Aldrich)) at 37 °C for 1 h. Sections were then rinsed in distilled water and dehydrated in a graded series of ethanol solutions (70% for 30 s, 96% for 30 s, 100% for 2 × 2 min, xylol 2 min) and mounted on glass coverslips.

### 2.2. Molecular Genetic Analysis

The complete set of DNA was isolated from the muscle or blood of all available patients using a standard protocol (muscle, n = 28, blood, n = 8). The complete mitochondrial genome of each examined patient was amplified in five overlapping segments per PCR reaction and subsequently purified using the GeneJet PCR Purification Kit (Thermo Scientific, Karlsruhe, Baden Württemberg, Germany), following the manufacturer’s instructions. In this study, a common deletion larger than 4977 bp, which cannot be amplified using conventional PCR techniques, was successfully amplified using a specialized technique used to magnify long DNA fragments. Long-range PCR enables the amplification of larger DNA fragments over an extended runtime, targeting the major arc of the mtDNA. This approach utilizes a single set of oligonucleotide primers to identify potential deletion regions within the mitochondrial genome. The forward primer (F) binds to nucleotides 8274–8305, while the backward primer (B) binds to nucleotides 13,720–13,642, flanking the 4977 bp region. When this region is deleted, it corresponds to the common deletion observed in patients with CPEO. The presence of mtDNA deletions was analyzed using a conventional long-range PCR method, as previously described [23,24].

#### Screening of the Common Deletion (CD)

A PCR was designed to detect a particular sequence in the mtDNA, which encompasses the 4977 bp common deletion (CD). The CD is flanked by a 13 bp direct repeat, with the 5′ end starting at nucleotide position 8469 in the ND4 region and ending at nucleotide position 13,446. The primers (forward primer: CCCTCTACCCCCTCTAGAGCCCACTGTAAAGC and reverse primer: GGCTTCCGGCTGCCAGGCCTTTAATGGGG) were so designed that they bind at either side of the region of CD. In case of persistence of the CD in the template, the PCR would amplify a DNA fragment of 470 bp, excluding the 4977 bp that the CD encompasses. The DNA fragment without the presence of the CD would not be amplified due to the very large size of the resulting fragment (470 + 4977 bp). The resulting amplified PCR product was directly sequenced using a normal protocol.

### 2.3. Statistical Analysis

Statistical analysis was performed using SPSS (version 16). To assess the level of common mtDNA deletion in patients with CPEO syndrome and analyze variations in clinical characteristics concerning age and gender, a one-way analysis of variance (ANOVA) was conducted. Additionally, basic statistical measures were used to evaluate the correlation between patients’ mean age and gender. A *p*-value of <0.05 was considered statistically significant.

## 3. Results

### 3.1. Clinical Characteristics

**Figure 1:**
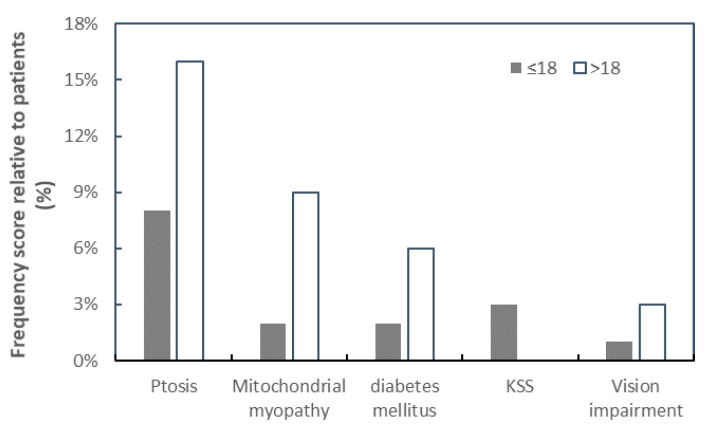
The relationship between frequency of patients with mtDNA common deletion and various clinical manifestations. The clinical characteristics in patients ≤ 18 years (closed square) and >18 years (open square) are plotted against the percentage frequency of mtDNA deletion. KSS was only observed to be manifested among the patient’s group ≤ 18 years.

The frequently observed clinical manifestation was ptosis (n = 20; 55.6%) followed by mitochondrial myopathy in (n = 9; 25%), diabetes mellitus type II (n = 6; 16.7%), and cardiac disorders in (n = 5; 13.9%). Detailed clinical data of patients are listed in Table 1. The thirty-six index patients in this study were grouped into two age groups: ≤18 years and >18 years (Table 2). The subsequent clinical symptoms were more frequently reported in patients older than 18 years, harboring the common deletion (Figure 1). Moreover, based on clinical characteristics, the patients were further divided into two sub-groups: patients with pure CPEO (n = 9) and patients harboring CPEO along with other symptoms (CPEO plus) (n = 27) (Table 2). It is interesting to note that among patients with CPEO plus, seven were harboring cardiac-related diseases (ischemic heart disease, arrhythmia, and bradycardia), together with muscle weakness. Endocrine-related disorders revealed among the index patients include hypercholesterolemia, lactose fructose intolerance, hyperlipidemia, and hypothyroidism.

### 3.2. Analytical Correlation Between Patients Age and mtDNA Deletion

**Figure 2:**
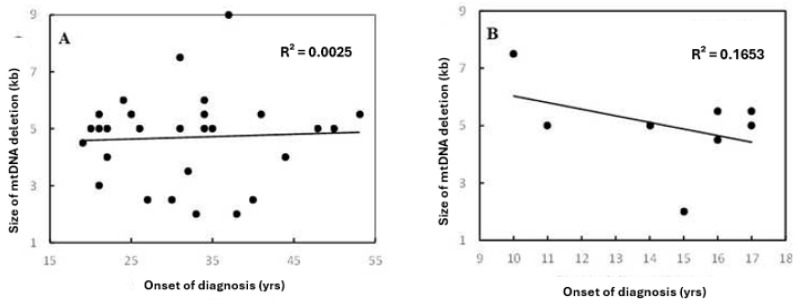
Correlation between patients’ onset of diagnosis and size of mtDNA deletion in (**A**) the total set for age group > 18 years and (**B**) datasets for age group ≤ 18 years. R^2^ indicates the strength of a linear relationship between the two parameters. No significant differences were found among both patients’ age groups, with the same frequency range. However, there were different coefficient correlations for each group.

**Figure 3:**
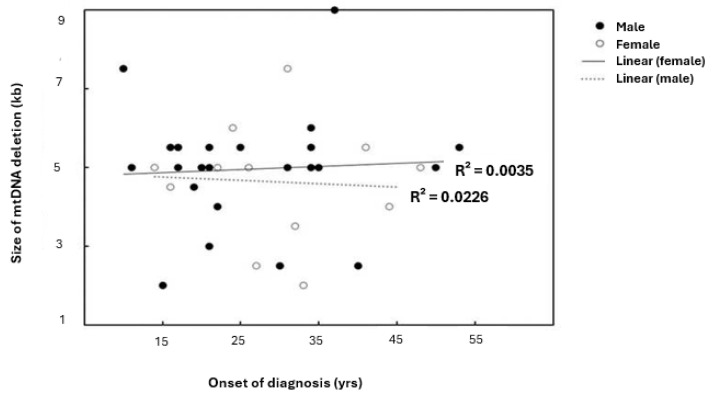
Linear correlation between patients’ onset of diagnosis and size of mtDNA deletion relative to gender. R^2^ indicates the strength of a linear relationship between the two parameters. Although there are no significant differences found among genders, with the same frequency range; the scatterplot and linear regression line showed the existence of variable coefficient correlations.

In patients older than 18 years, a positive correlation between the age of onset of patients and the size of mtDNA deletion was observed (coefficient correlation, R = 0.050) (Figure 2A). With ages ≤ 18 years, the relationship was negatively correlated (R = −0.0826) (Figure 2B). This correlation was more prominent in females (Figure 3). Furthermore, it was observed that patients harboring KSS had higher frequency scores among patients diagnosed at an onset age ≤ 18 years, which was associated with the presence of a CD.

### 3.3. Comparative Analysis of Age at Disease Onset and Clinical Symptoms

The clinical features range from muscle weakness, cardiac-related disorders, and neurological disorders, with onset age diagnosed mostly at a later age, as observed in previous studies [25]. Elderly patients harboring the CPEO plus had a higher frequency of common deletions in the mtDNA, with more variability among female patients. The age of disease onset in 26 patients (63.4%) was observed to be before the age of thirty, and the patient with KSS manifested before twenty years of age. One patient with CPEO plus exhibited seizures at the onset age of 53 years. A lower mean age of disease onset was observed among female patients as compared to male patients. The most critically affected patients had a mean age of 43.4 years (ranging from 27–74 years of age), with an onset age of 11 years old.

### 3.4. Genetics Evaluations Reveal the Presence of the mtDNA 4977 bp Common Deletion (CD)

Using the conventional PCR program, a common deletion of 4977 bp along the entire mitochondrial genome was detected, with designed primer pairs corresponding to both 5’ and 3’ breakpoints of the mtDNA deletion. In the presence of this large-sized deletion (4977 bp) on the mtDNA, amplification of fragments will occur, and the PCR product size will be 470 bp. However, amplification of fragments will not occur without this deletion and thus will be 5447 bp (470 bp + 4977 bp) in product length. A single mtDNA deletion was identified in all patients. The length of deletions ranged from ~2 kb to ~9 kb, and the common 4977 bp deletion (CD) was identified in 11 patients (five males and six females). The age groups of patients harboring this common deletion are shown in Table 3.

### 3.5. Histological Evaluation Reveals mtDNA Disorder

**Figure 4:**
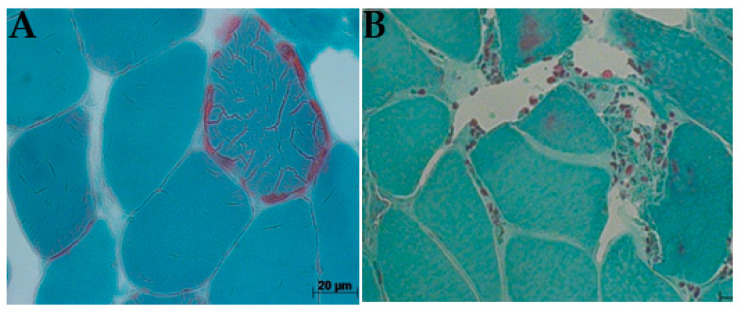
Ragged-red fibre (arrows), biopsy from patients P17 (**A**) and P7 (**B**): P17 showed a typical ragged red fiber with red thread-like speckling of diseased mitochondrial in patients with CPEO plus due to proliferation of abnormal mitochondrial; P7 exhibited small, spotted abundance of intervening collagenous deposits caused by muscle myopathy in patient harboring CPEO with visual impairment (Gomori trichrome × 200).

**Figure 5:**
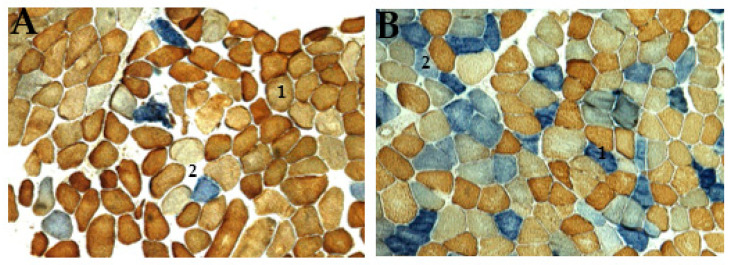
Histochemical activity of patient’s skeletal muscle tissue stained with COX (brown) and SDH (blue) enzymes, often found in mitochondrial diseases. (**A**) showing a typical distribution of brown mosaic pattern (1, normal COX activities), light or white pattern (2, no COX activity), and blue coloration (Asterix, increased SDH activity with no COX activity); (**B**) abnormal distribution of mitochondrial are stained with two distinctive blue colorations; dark blue (1, COX-negative fiber/strong SDH activity), and light blue (2, mild COX deficiency/weak SDH activity). This pattern of staining indicates defects in mtDNA in patients (original magnification ×100).

For each muscle tissue, staining was evaluated, and the percentage of RRFs and COX/SDH deficiency (COX-negative, SDH-positive) fibers in each muscle sample was determined by counting 200–500 muscle fibers on each tissue section. This was conducted using electron microscopy under a low-power field magnification (×10 objective), separating both normal and abnormal pathological features. Gomori trichrome staining showed connective tissue, dark white, and muscle fibers, blue, with a red coloration, which indicates the presence of mitochondria [26]. Examining the histology of the cryosection muscle tissue of the patient showed the accumulation of mitochondrial subsarcolemmal, an indication of the RRF (Figure 4), and COX-deficiency fibers (Figure 5). These RRFs have features that reveal the usual proliferation of mitochondria, which could be caused by COX deficiencies.

## 4. Discussion

CPEO is known to be the most common clinical presentation associated with a single, large-scale mtDNA deletion, characterized by eyelid ptosis, visual impairments caused by ocular myopathy, and ophthalmoparesis [11,14]. This is in comparison with the genetic effects, common mtDNA deletions commonly found in CPEO patients [8,27]. The present analysis showed that ptosis is invariably associated with patients harboring single mtDNA deletions in all age groups, irrespective of gender (Figure 4). This was in line with a previous study reporting ptosis as the most clinically significant symptom associated with CPEO [28].

Studies have frequently reported a common 4977 bp deletion (CD) in CPEO and KSS patients [29,30]. Other frequently identified single deletions associated with CPEO include 834 bp [31], 7400 bp [32], and 4867 bp [33] deletions. In the present study, the CD was identified in 30.6% of patients, more frequently (36.4%) in elderly patients (>18 years of age group). This is consistent with the earlier report [34]. It is imperative to note that these findings could be related to CPEO patients exclusively or the tissue type present at the time of biopsy, concerning previous reports, which stated that CD type could hardly be detected in tissues (heart and skeletal muscle) amongst younger patients under 20 years of age [12,35].

Patients with KSS had their onset age of disease of less than 18 years, exhibited short stature, and had a higher frequency of large-sized mtDNA deletions compared to other clinical diagnoses. These observations were in line with previous findings on patients with mitochondrial disorders [19]. Most research findings usually target the location and size of mtDNA mutation; contrary to this work, we studied the mtDNA deletion and how it relates to any changes in the skeletal muscle tissues and clinical presentation. This has presented a limitation to this work. The presentation of KSS at an early age of 18–20 years, which cannot be revealed in one-third of the patients used in this study, also showed a limit to this study. However, a study carried out by Mancuso (2015) proposed a new diagnostic criterion called the “KSS spectrum” to properly diagnose patients harboring KSS [36]. This spectrum detailed clinical characteristics like tremor, cardiomyopathy, and short stature, which are revealed to features observed in KSS. These clinical features were also observed in the KSS patients in this present study. The histochemical study of mitochondrial enzyme activities associated with ophthalmoplegia in patients’ tissues would have confirmed any disorder in the mitochondrial DNA. Unfortunately, we did not have the capacity to perform any of such enzyme activities in this study.

The age group younger than 18 years had the highest mean score relative to mtDNA deletion size, while in patients older than 18 years, the deletion mean size varied much less than the younger age group. Hence, the age group (≤18 years) and its more consistent relative deletion mean size should make predictions of patients harboring mtDNA deletions more dependable than variabilities in other age groups (Table 2). More than 25% of patients harboring the CD were females. Interestingly, the CD was identified more consistently among males. The present study further revealed that female patients with CPEO are more variable, especially at lower CD levels, although their median value was closely associated with male patients within more than half the size of the overall patients. This implies that a majority of the study population harbors CD. Although a determining factor concerning mitochondrial diseases in various research has experienced a focus mainly on molecular diagnosis and studies [36]; genetic study alone is not sufficient to ascertain any changes that may occur in the clinical manifestations among patients. The percentage of subsarcolemmal mitochondrial accumulation on muscle biopsies also plays a crucial additional role in determining any disease conditions caused by dysfunctional mitochondria.

Ptosis and mitochondrial myopathy were the major clinical diagnoses related to CPEO, with all other diagnostic factors manifesting primarily at ages > 18 years. Ages ≤ 18 years seem to be the most consistent group of patients associated with KSS and other clinical phenotypes, although a higher frequency score for ptosis amongst age groups > 18 years was observed. The present study established that the risk factor, ptosis, is an early sign of CPEO, suggesting that mitochondrial mutations in patients may be initiated at an early stage of CPEO. This was consistent with mitochondrial disease onset before the age of 18 [19]. The photomicrograph revealed both RRF and COX deficiency fibers (Figure 4 and Figure 5) in all but one patient. In agreement with previous studies, this research revealed higher frequencies of COX deficiency fibers, much more than the number of RRF present in a tissue biopsy, and an increased tendency of COX deficiency fibers with increasing age among patients.

There are a few limitations that were observed within the confines of this present study. Among these is the small sample size, limiting any significance that may have existed within the patient’s group, and the limited number of clinical diagnoses presented by patients. Although direct sequencing was performed from products of conventional PCR products, they were not enough to detect any changes in nucleotides, sizes, or specific locations of point mutations that may occur in the mtDNA.

## 5. Conclusions

Single mtDNA deletions are consistently linked to CPEO and CPEO plus, with the common 4977 bp deletion being a frequent cause of phenotype manifestation. These deletions appear to be more commonly associated with older patients. There was a tendency for an increased frequency of COX-deficient fibers with age in patients harboring the 4977 bp deletion. This deletion was also found to be associated with elderly patients with CPEO. Using the sequential COX/SDH staining technique, a tissue sample showed a normal brown mosaic pattern of muscle fibers (COX-positive). However, further genetic screening of this tissue revealed a large-scale mtDNA deletion, which otherwise would have been overlooked. However, due to the limitations of this study, particularly the small sample size, further longitudinal research is necessary to validate and extend these findings. Such research could focus on identifying early risk factors, improving disease monitoring, and establishing risk factors that predispose individuals to developing specific diseases.

## Figures and Tables

**Table 1:** Epidemiological, clinical, and molecular characteristics of patients.

P	G	A (yrs)	O (yrs)	Clinical Symptoms	DS (kb)
1	F	47	20	CPEO, ptosis, LFI, CK	5.0
2	F	24	16	CPEO	5.5
3	F	42	31	CPEO, KSS	5.0
4	F	19	19	CPEO	4.5
5	F	29	21	CPEO, ptosis, Mm	5.0
6	F	32	17	CPEO, ptosis, myopathy, KSS	5.5
7	F	43	30	CPEO, ptosis, perinatal hypoxia	2.5
8	F	30	11	CPEO, ptosis, UTI, IDA	5.0
9	F	34	34	CPEO	6.0
10	F	21	21	CPEO, VI	5.5
11	F	37	37	CPEO	9.0
12	F	34	34	CPEO	5.5
13	F	27	21	CPEO, subacute dysphagia, DM2 ptosis, myopathy,	3.0
14	F	54	50	CPEO, ptosis, Mm, glaucoma, UTI	5.0
15	F	61	34	CPEO	5.0
16	F	74	22	CPEO, ptosis, DM2 absolute arrhythmia, IHD	4.0
17	F	49	40	CPEO, ptosis, DM2 absolute arrhythmia, IHD	2.5
18	F	14	10	CPEO, ptosis	7.5
19	F	55	25	CPEO, ptosis, cataract, DE	5.5
20	F	53	53	CPEO, Mm	5.5
21	F	49	35	CPEO, ptosis, migraine	5.0
22	F	50	15	CPEO, ptosis	2.0
23	F	27	17	CPEO, Ptosis, VI, DM2	5.0
24	F	40	38	CPEO, ptosis, Mm	2.0
25	F	36	27	CPEO	2.5
26	M	15	14	CPEO	5.0
27	M	65	33	CPEO, Mm	2.0
28	M	38	31	CPEO, mitochondrial myopathy	7.5
29	M	25	24	CPEO, myopathy, intracerebellar hemorrhage	6.0
30	M	33	26	CPEO	5.0
31	M	42	22	CPEO, ptosis	5.0
32	M	32	32	CPEO, mitochondrial myopathy	3.5
33	M	66	48	CPEO, ptosis, DM, IHD, hyperlipidemia	5.0
34	M	71	16	CPEO, ptosis, DM, Mm	4.5
35	M	49	41	CPEO, ptosis, arrhythmia, hyperlipidemia, arterial hypertonia	5.5
36	M	73	44	CPEO, ptosis, diabetes mellitus type 2, bradycardia, arterial hypertonia	4.0

P: patients, G: gender, F: female, M: male, A: age at biopsy, O: onset of biopsy, DS: deletion size, mtDNA: mitochondrial DNA, CPEO: chronic progressive external ophthalmoplegia, KSS: Kearns–Sayre Syndrome, kb: kilobase, VI: vision impairment, DM2: diabetes mellitus type 2, Mm: mitochondrial myopathy, UTI: urinary tract infection, IDA: iron deficiency anemia, IHD: ischemic heart disease, LFI: lactose and fructose intolerance, CK: chronic keratopathy, DE: depressive episode.

**Table 2:** Genetic features of mtDNA deletion in patients according to age groups.

Age Group (Years)	No. of Patients	Mean Age (±SD)	Range of Deletion (kb)	Frequency of CD
≤18	8	14.50 (±5.0)	2.0–7.5	2 (18.18%)
>18	28	31.90 (±9.5)	2.0–9.0	9 (81.82%)

SD: standard deviation, kb: kilobase, CD: common deletion.

**Table 3:** Relationships between mtDNA deletion and major clinical symptoms in patients with and without common deletion.

Patients	No. of Patients	With CD	*p*-Value	Without CD	*p*-Value
Total	36	11 (30.56%)		25 (69.44%)	
Ptosis	20	8 (40.00%)	0.013 *	12 (60.00%)	0.032 *
Mm	9	3 (33.33%)	0.286	6 (66.67%)	0.019 *
VI	4	2 (50.00%)	0.337	2 (50.00%)	0.668
DM	6	3 (50.00%)	0.354	3 (50.00%)	0.415
KSS	2	2 (5.56%)	0.038 *	No value	

* *p* < 0.05, statistically significant, Mm: mitochondrial myopathy, VI: vision impairment, DM: diabetes mellitus, CD: common deletion.

## Data Availability

The datasets analyzed during the current study are contained within this article, and available from the corresponding author upon kind request.

## Abbreviations

The following abbreviations are used in this manuscript:

mtDNA	mitochondrial DNA
CPEO	chronic progressive external ophthalmoplegia
KSS	Kearns–Sayre Syndrome
kb	kilobase
P	patients
G	gender
F	female
M	male
A	age at biopsy
O	onset of biopsy
DS	deletion size
kb	kilobase
VI	vision impairment
DM2	diabetes mellitus type 2
Mm	mitochondrial myopathy
UTI	urinary tract infection
IDA	iron deficiency anemia
IHD	ischemic heart disease
LFI	lactose and fructose intolerance
CK	chronic keratopathy
DE	depressive episode
CD	common deletion
